# 

**Published:** 1880-12

**Authors:** 


### INDEX TO VOLUME XLI.

A.

Page.

Addison's Disease, T. F. Rochester, M.D. .... 318
Address, abstract of, by Dr. DeLaskie Miller, to Alumni Association, Rush College, Feb. 25, 1880 .... 586
Air, organic matter in .... 512
American Medical Association; 31st annual meeting, 1880 .... 71
Anatomy, cerebral, simplified .. 449
Anæmia, transfusion in .... 314
Andrews, E., M.D., Prof. Clay's new cancer cure .... 141
Aneurism of the arch of the aorta cured by rest, etc., by R. S. Sutton, M.D .... 663
Announcements for the months, 112, 224, 336, 448
Antiseptics in ophthalmic surgery .... 6
Asbestos for splints .... 332
Association, American Medical, 31st annual session, 1880 .... 71
Asthma, the treatment of, Dr. J. B. Berkart .... 656
Asthma, iodide of ethyl in, by D. R. Brower, M.D .... 68
Atkinson, I. E., M.D., a clinical lecture upon cutaneous epithelioma .... 188
"Atrophy, simple," or wasting, Dr. Lewis Starr, on .... 648
Atresia of the genital passages of women, by Edw. W. Jenks, M.D .... 225

B.

Baking powders, examination of, by R. S. G. Paton .... 264
Battey's operation, by A. R. Jackson, M.D .... 456
Bladder, stone in the, by Dr. R. F. Weir .... 1
Board of Health of Michigan .. 162
Board of Health of Michigan, report .... 479

Page

Book Notices and Reviews .... 102, 176, 285, 389, 488
Brophy, T. W., caries of superior maxilla .... 582
Book Notices and Reviews .... 602
Bromide of ethyl a disappointing as well as dangerous anæsthetic, by J. C. Moore, M.D. 155
Brower, D. R., M.D., iodide of ethyl in asthma .... 68
Brower, D. R., M.D., hyoscyamia .... 261

C.

Calculi renal, R. S. Weir, M.D .. 317
Cancer cure, Prof. Clay's new, by E. Andrews, M.D .... 141
Cancer, case of uterine, C. F. Parkes, M.D .... 373
Caries of superior maxilla, T.W. Brophy, M.D .... 582
Coskery, O. J., M.D., a case of fracture of the cervical spine 260
Causes of sickness in Michigan. 280
Cerebral rheumatism, by P. O'Connell, M.D .... 54
Chancre of the meatus and urethra in the male, by J. N. Hyde, M.D .... 113
Cholera infantum, oxalate of cerium in treatment of .... 592
Chloral, the treatment of gonorrhœa by the injection of .... 187
Chloral, some observations on the cutaneous eruptions attending the use of, by Mr. Mayor .... 271
Church, Theo. B. Obituary ... 559
Cirrhotic kidney, fatal case, etc. Wm Russell, M.B .... 322
Clevenger, S. V., M.D., cerebral anatomy simplified .... 49
Clinics .... 224, 336, 448
Conjunctivitis, epidemic, by Lester Curtis, M.D .... 159
Consumption, contagion of, J. T. Whitaker, M.D .... 328

PAGE.
Contagion of consumption, J. T. Whitaker, M.D. .......... 328
Correspondence, domestic, Philadelphia letter.............. 96
Correspondence, domestic, Boston letter.................. 171
Correspondence, domestic, New York letter................ 383
Correspondence, domestic. 484, 593
Correspondence, foreign, Dr. A. Lagorio, letter from Italy.... 165
Correspondence, Foreign...281, 379
Coryza, acute, remedy for, (M. Yron, *Jour. de Méd.*, May, 1880.)...................... 161
Cremation, by Dr. Wm. Porter. 421
Curtis, Lester, M.D., epidemic conjunctivitis.............. 159

D.

Davis, Frank H., obituary..... 333
Diabetometer, the............. 498
Diagnosis, difficulties of....... 509
Difficulties of diagnosis ...... 509
Dilatation of the stomach...... 529
Diphtheria and croup; tracheotomy in; E W. Lee, M.D., and Chr. Fenger, M.D ...... 337
Diphtheritic exudation, effects of inoculating lower animals with.................... 504
Domestic correspondence, New York letter................ 383
Domestic correspondence...484, 593
Duboisia, case of poisoning by, E. L. Holmes, M.D.......... 475

E.

Earle, C. W., M.D., Leucasmus, or Vitiligo................ 255
Editorials, American Medical Association................ 109
Editorials, Medical education, the Northwest still in the lead 111
Editorial, American Medical College Association......396, 499
Enteritis, Membranous........ 532
Epidemic conjunctivitis, by Lester Curtis, M.D............... 159
Epithelioma, cutaneous, a clinical lecture upon, by I. E. Atkinson, M.D................ 188
Epithelioma of upper end of œsophagus................. 522
Eruptions; some observations on the cutaneous, attending the use of chloral, by Mr. Mayor ........................ 271
Etheridge, Dr. J. H., on the management of the uterus after parturition.................. 127

PAGE.
Ethyl, the bromide of, a disappointing, as well as a dangerous anæsthetic ............. 155
Etiology and prophylaxis of premature baldness............ 284

F.

Fenger, Dr. Chr., and E. W. Lee, select topics in modern surgery ........................ 7
Foreign correspondence....281, 379
Fracture of the cervical spine, a case of, by O. J. Coskery, M.D. 260

G.

Genital passages of women, atresia of, by E. W. Jenks, M.D.. 225
Girls, treatment of leucorrhœa in little.................... 327
Glycerine in flatulence, acidity and pyrosis, Sidney Ringer, M.D........................ 325
Goldsberry, J. A., M.D., hysteria. 35
Gonorrhœa, treatment of, by injection of chloral............ 187
Gonorrhœal ophthalmia, a novel mode of treatment of........ 538
Goodrich, Dr. Calvin G., obituary ........................ 669
Gynæcology, selections........ 543

H.

Heat, the regulation of body... 535
Holmes, E. L., M.D., case of poisoning by duboisia....... 475
Hospitals of Paris............ 471
Hotz, F. C., M.D., a fatal case of acute suppurative otitis...... 150
Hotz, F. C., M.D., aural complaints in consequence of malaria.................... 472
Hyde, J. N., M.D., on chancre of the meatus and urethra in the male...................... 113
Hydrophobia, new remedy for.. 221
Hygiene of genital organs of young children, genital irritation, R. Park, M.D......... 561
Hyoscyamia, by D. R. Brower, M.D........................ 261
Hysteria, by J. A. Goldsberry, M.D........................ 35
Hysterical muscular contractions, W. T. Speaker, M.D... 468

I.

Incompatibility of pepsin with certain other drugs, Kretzschmar ...................... 101
Indican in health and disease, H. N. Heineman, M.D........... 318

PAGE.
Infants, the "summer complaint" of, by Dr. J. Lewis Smith ..................... 212
Ingals, E., M.D., how to feed the sick ......... .............. 269
Ingals, E. F., M.D., treatment of diseases of the larynx........ 348
Inoculating lower animals with diphtheritic exudation, effects of ........................ 504
Iodide of ethyl in asthma, by D. R. Brower, M.D ............. 68
Items, writers' palsy.......... 670

J.

Jackson, A. R., M.D., Battey's operation.................. 456
Jenks, E. W., M.D., atresia of the genital passages of women... 225

K.

Kidney, cirrhotic, fatal case etc., Wm. Russell, M.B... ...... 322

L.

Larynx, treatment of diseases of, by E. F. Ingals, M.D......... 348
Lee, Dr. E. W., and Christian Fenger, select topics in modern surgery ................. 7
Letter, Philadelphia, domestic correspondence.............. 96
Leucasmus, or vitiligo, by C. W. Earle, M.D....... ...... 255
Leucorrhœa, treatment of, in little girls.................. 327

M.

Malaria a cause of aural complaints, F. C. Hotz, M.D...... 472
Meatus and urethra of the male, on chancre of, by J. N. Hyde, M.D....................... 113
Medical, American, Association, 31st annual session, 1880..... 71
Membranous enteritis.......... 532
Miller, DeLaskie, M.D., abstract of address delivered before Alumni Association of Rush College ..................... 586
Moore, J. C., M.D., the bromide of ethyl a disappointing as well as dangerous anæsthetic 155
Movement, as a therapeutic agent, J. K. Spender, M.D.... 305
Muscular contractions, hysterical, W. T. Speaker, M.D...... 468

N.

Natural selection in diseases; H. D. Valin, M.D............ 579

PAGE.
Nephritis, a clinic on cases of, F. Delafield, M.D............ 317
Notes from private practice.... 590

O.

Obituary, Frank H. Davis, M.D. 333, 667
Obituary, T. B. Church....... 559
Obituary, Dr. Calvin G. Goodrich...... ... ............. 669
Obstetrics, selections, a case of spontaneous version......... 552
O'Connell, P., M.D., cerebral rheumatism.. ............. 54
Œsophagus, epithelioma of upper end of... .............. 522
Olecranon, operation for united fracture of the, by opening joint and removing fragments of ulna, Mr. Wm. Rose...... 431
Ophthalmic surgery, antiseptics in ........................ 6
Ophthalmia, gonorrhœal, a novel mode of treatment of.. 538
Otitis, a fatal case of acute suppurative, by F. C. Hotz, M.D . 150
Ovarian cysts, complications, T. P. Seeley, M.D............ 570

P.

Parkes, C. T., M.D., a case of uterine cancer............... 373
Park, R., M.D., genital irritation, hygiene of genital organs of young children......... .. . 561
Parturition, on the management of the uterus after, by Dr. J. H. Etheridge.... ........... 127
Paton, R. S. G., examination of baking powders............. 264
Pathology of tubercle, by J. M. DaCosta, M.D .. ........... 295
Pathology, renal and urinary... 433
Pepsin, its incompatibility with certain drugs............... 101
Perityphylitis, Dr. H. B. Sands on .............. ......... 619
"Perineal section," treatment of urethral stricture by, by A. L. Ranney, M.D ... ........... 361
Pregnancy, pomade for the spots of, *L'Union Médicale*, No. 59. 175
Prolapse of bladder in a case of labor, H. D. Valin, M.D .... 477
Prophylactic inoculation summed up, H. D. Valin, M.D.... 461
Prussic acid and a new alkaloid in tobacco smoke............ 532

Q.

PAGE.
Quinquinia, why it should be used by country practitioners, by J. S. Whitmire, M.D...... 145

R.

Renal calculi, R. S. Weier, M.D. 317
Reports, society, Aurora Medical Society.................. 600
Report of Michigan State Board of Health................. 162
Report of the committee appointed to recommend a plan of medical registration of medical colleges of this country ..................... 203
Reviews and Book Notices.... 176, 285, 389, 488, 602
Rheumatism, cerebral, by P. O'Connell, M.D................ 54
Rheumatism, chronic, Dr. Wm. Pepper, on................... 619
Ringer, Sidney, M.D., glycerine in acidity, flatulence and pyrosis ......................... 325

S.

Sanitary Science; list of books valuable for candidates in examination of............... 443
Sayre's treatment for spinal curvature, improvement of.. 313
Selections; Dr. H. B. Sands on perityphlitis................ 605
Selections; Dr. Wm. Pepper on chronic rheumatism......... 619
Selections; Dr. R. D. Fox on treatment of sprains......... 644
Selections; Dr. Louis Starr on wasting, or "simple atrophy." 648
Selections; Dr. J. R. Berkart, on treatment of asthma ..... 656
Selections; Dr. R. S. Sutton, on aneurism of arch of the aorta. 663
Sick, how to feed the, by E. Ingals, M.D.................. 269
Smith, Dr. J. Lewis, on the "summer complaint" of infants ........................ 212
Society meetings......224, 336, 448
Society meetings; Indiana, Illinois and Kentucky Tri-State Medical Society ............ 555
Society meetings; clinics...560, 672
Society reports................ 600
Spinal curvature, improvement of Sayre's treatment for...... 313
Spine, a case of fracture of the cervical, by O. J. Coskery, M.D. 260
Splints, asbestos for........... 332
Sprains, Dr. R. Dacre Fox on treatment of................ 645
Stomach, dilatation of......... 529
Stone in the bladder, by Dr. R. F. Weir ..................... 1
Strangulated vermiform appendix; operation, death, remarks 518
Stricture, treatment of urethral, by "perineal section," O. S. Ranney, M.D................. 361
Summary, therapeutics....... 222
"Summer complaint" of infants, by J. S. Smith, M.D.... 212
Superfœtation, a case of, D. A. Walden, M.D................ 69
Superior maxilla, caries of, T. W. Brophy, M.D............. 582
Surgery, select topics of modern, by Drs. E. W. Lee and Chr. Fenger...................... 7
Surgery; selections............ 536

T.

Therapeutics, a summary of.... 222
Therapeutics, selections....... 553
Thermometric bureau......... 427
Tracheotomy in croup and diphtheria, E. W. Lee, M.D., and Chr. Fenger, M.D............ 337
Transfusion in anæmia........ 314
Translations; nature and pathogeny of malignant jaundice, by A. Mathieu............... 404
Translations.................. 515
Treatment of diseases of the larynx, by E. F. Ingals, M.D. 348
Trephining of skull of lunatic on old head injury, complete recovery ................... 536
Tubercle, pathology of, J. M. DaCosta, M.D................ 295
Typhoid fever, specific agent of, Prof. Klebs................. 517

U.

Urethra, on chancre of the, and meatus in the male, by J. N. Hyde, M.D.................. 113
Urethritis, chronic, treated with injections of water, saturated with tincture of iodine...... 175
Uterus, on the management of, after parturition, by J. H. Etheridge, M.D.............. 127

V.

Valin, H. D., M.D., prolapse of bladder in case of labor...... 497

PAGE.
Vitiligo, leucasmus, or, by C. W. Earle, M.D. .................. 255

W.

Walden, D. A., M.D., a case of superfœtation... ........... 69
Weir, Dr. R. F., stone in the bladder .................... 1

PAGE.
Wintergreen, essence of, as an antiseptic, M. Perier, (*Jour. de Méd.*, May, 1880.) ........ 161

Whitmire, Dr. J. S., quinquinia, why it should be used by country practitioners........ 145

### SOCIETY MEETINGS.

Chicago Medical Society—Mondays, Dec. 1 and 15.
West Chicago Medical Society—Mondays, Dec. 8 and 22.
Biological Society—Wednesday, Dec. 3.

### CLINICS.

MONDAY.

Eye and Ear Infirmary—2 p. m., Ophthalmological, by Prof. Holmes; 3 p. m., Otological, by Prof. Jones.

Mercy Hospital—2 p. m., Surgical, by Prof. Andrews.

Rush Medical College—2 p. m., Dermatological and Venereal, by Prof. Hyde.

Woman's Medical College—2 p. m., Dermatological and Venereal, by Prof. Maynard; 3 p. m., Diseases of the Chest, Prof. Ingals.

TUESDAY.

Cook County Hospital—2 to 4 p. m., Medical and Surgical Clinics.

Mercy Hospital—2 p. m., Medical, by Prof. Quine.

WEDNESDAY.

Chicago Medical College—2 p. m., Eye and Ear, by Prof. Jones.

Rush Medical College—2 p. m., Medical, by Dr. Bridge; 3 p. m., Ophthalmological and Otological, by Prof. Holmes; 3:30 to 4:30 p. m., Diseases of the Chest, by Dr. E. Fletcher Ingals.

THURSDAY.

Chicago Medical College—2 p. m., Gynæcological, by Prof. Jenks.

Rush Medical College—2 p. m., Diseases of Children, by Dr. Knox; 3 p. m., Diseases of the Nervous System, by Prof. Lyman.

Eye and Ear Infirmary—2 p. m., Ophthalmological, by Dr. Hotz.

Woman's Medical College—3 p. m., Surgical, by Prof. Owens.

FRIDAY.

Cook County Hospital—2 to 4 p. m., Medical and Surgical Clinics.

Mercy Hospital—2 p. m., Medical, by Prof. Davis.

SATURDAY.

Rush Medical College—2 p. m., Surgical, by Prof. Gunn; 3 p. m., Orthopœdic, by Prof. Owens.

Chicago Medical College—2 p. m., Surgical, by Prof. Isham; 3 p. m., Neurological, by Prof. Jewell.

Woman's Medical College—11 a. m., Ophthalmological, by Prof. Montgomery; 2 p. m., Gynæcological, by Prof. Fitch.

Daily Clinics, from 2 to 4 p. m., at the Central Free Dispensary, and at the South Side Dispensary.

